# Primary cervical spine carcinoid tumor in a woman with arm paresthesias and weakness: a case report

**DOI:** 10.1186/1752-1947-7-214

**Published:** 2013-08-23

**Authors:** Mohan Narayanan, Daniel Serban, Gabriel C Tender

**Affiliations:** 1 SOM Office of Student Affairs, 2020 Gravier St., 7th floor, New Orleans, LA 70112, USA; 2 Department of Neurosurgery, “Bagdasar-Arseni” Hospital, Sos. Berceni, nr. 12, sector 4, Bucharest 041915, Romania; 3 Department of Neurosurgery, Louisiana State University, 2020 Gravier Street, Suite 744, New Orleans, LA, USA

**Keywords:** Carcinoid, Cervical spine, Neuroendocrine, Stenosis

## Abstract

**Introduction:**

Carcinoid tumors are neuroendocrine neoplasms derived from the enterochromaffin cells. Central nervous system involvement is rare and has been reported either as metastases to the brain and spine or primary tumors involving the sacrococcygeal spine. We report the first case of a primary carcinoid tumor of the cervical spine.

**Case presentation:**

A 50-year-old African-American woman presented with a 4-month history of numbness, paresthesias, and mild left-hand weakness. Magnetic resonance imaging of her cervical spine revealed a homogenously enhancing extradural mass, indenting the cervical cord and expanding the left neural foramen at C7–T1. A C7 corpectomy, en bloc resection of the tumor, and anterior C6–T1 fusion were performed to decompress the spinal cord and nerves and provide stability. Postoperative histopathologic examination and immunohistochemical analysis were consistent with carcinoid tumor. There has been no recurrence at the 6-year follow-up visit.

**Conclusions:**

Primary cervical carcinoid tumor is extremely rare, but should be included in the differential diagnosis of enhancing expansile extradural masses compressing the spinal cord and nerves. Surgical resection may provide a definitive cure.

## Introduction

Carcinoids are the most common neuroendocrine tumors [1], deriving from the enterochromaffin cells of the gut and bronchi. Originally believed to behave in a benign fashion, analysis of a large group of patients showed a 67.2% 5-year survival and a 12.9% incidence of metastases at the time of diagnosis [2]. Central nervous system metastases are infrequent and involve either the brain [3] or the spinal column [4-9]. Primary carcinoid tumors of the spine are extremely rare and have been described in the sacrum [10,11] and coccyx [12]. We report the first case, to the best of our knowledge, of a primary carcinoid tumor in the cervical spine.

## Case presentation

A 50-year-old African-American woman presented with a 4-month history of pain, paresthesias, and mild weakness in her left upper extremity. Her past medical history was significant for hypertension and borderline diabetes mellitus. Her family history was positive for breast cancer (sister), prostate cancer (father), and ovarian cancer (two paternal aunts). The patient had a five-pack-year history of smoking cigarettes, but had stopped smoking 7 years prior to presentation. Her review of systems was negative for any hormonal imbalance. A neurological examination revealed hypesthesia and mild weakness in her left C8 spinal nerve distribution, as well as mild hyperreflexia and a Babinski’s sign in the left lower extremity. Preoperative laboratory tests (including complete blood count, complete metabolic panel, and coagulation studies) showed borderline anemia, hypokalemia, and hypoalbuminemia (all asymptomatic).

**Figure 1:**
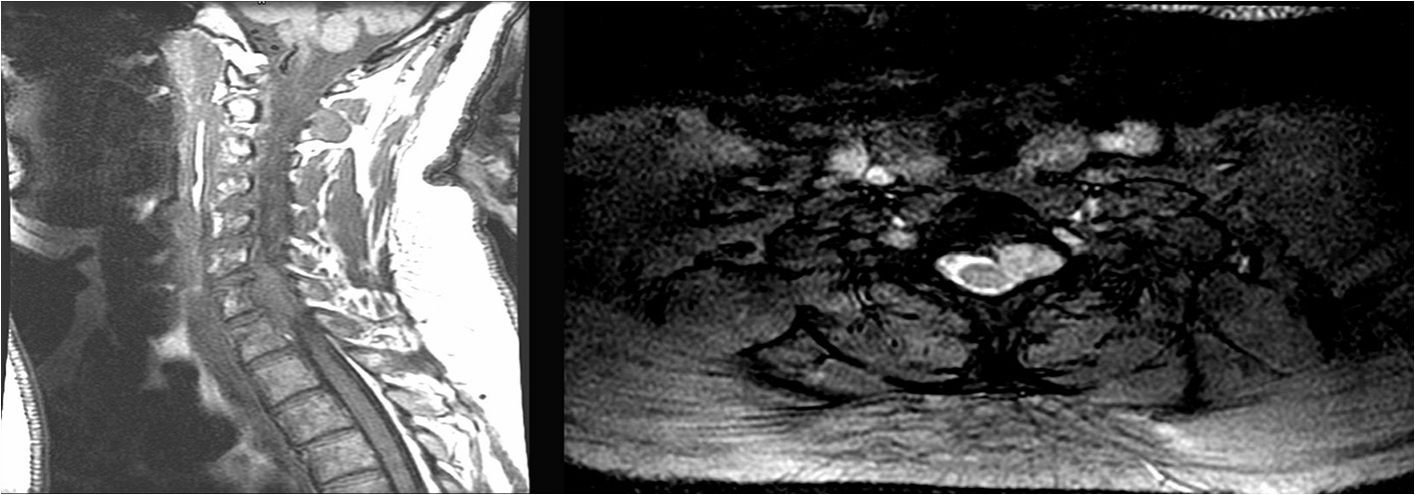
**Preoperative magnetic resonance imaging of the cervical spine.***Left*, T1-weighted sagittal magnetic resonance imaging (MRI) illustrating the isointense extradural mass, centered behind the vertebral body of C7 and indenting the spinal cord. *Right*, T2-weighted axial MRI illustrating the hyperintense and relatively well-demarcated extradural mass expanding the left neural foramen.

T1-weighted magnetic resonance imaging (MRI) of the cervical spine (Figure 1, left) revealed an extradural mass behind the vertebral body of C7, eccentric to the left, indenting the spinal cord, and enhancing homogenously with contrast. T2-weighted MRI showed the hyperintense extradural mass of 2.7×1.8×1.6cm, expanding the left neural foramen at C7–T1 (Figure 1, right). A full metastatic work-up, including computed tomography (CT) imaging of her chest, abdomen, and pelvis, revealed no potential primary cancer. Surgical intervention was recommended to obtain tissue for diagnosis and decompress the neural elements.

Under general anesthesia, a standard anterior cervical approach exposed the C6 and C7 discs, along with the adjacent C5 and T1 vertebral bodies. A C6–7 and C7–T1 discectomy was followed by a C7 corpectomy, started slightly right of the midline and extended laterally to the left. The tumor was contiguous with the bone, but much softer, and it compressed the dura mater, but without any obvious invasion and with a clear separating plane. Frozen section pathologic analysis yielded a preliminary diagnosis of metastatic undifferentiated small cell carcinoma, with probable origin from the lung. A gross total resection of the tumor was performed. Due to the extensive and destabilizing bony removal necessary for tumor resection, a C6–T1 instrumented fixation was deemed necessary and performed in a standard fashion. The patient tolerated the procedure without complications.

**Figure 2:**
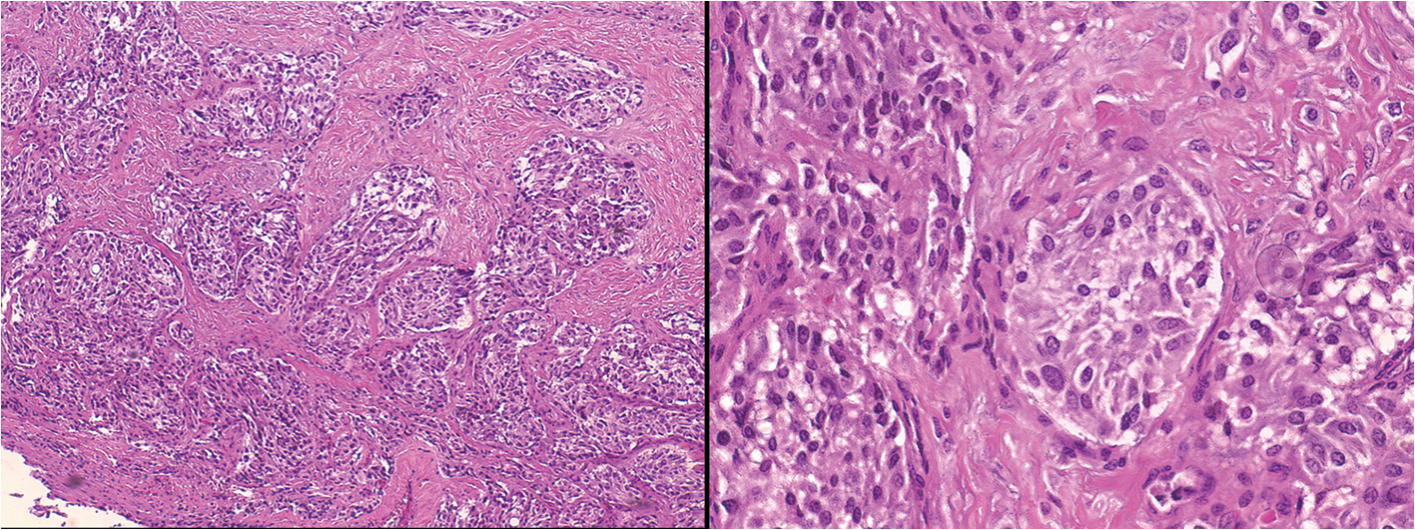
**Histopathology.** Photomicrographs of the specimen showing sheet-like proliferation of neuroendocrine cells, which suggested the diagnosis of carcinoid tumor (hematoxylin and eosin, original magnification ×10, *left*, and ×40, *right*).

**Figure 3:**
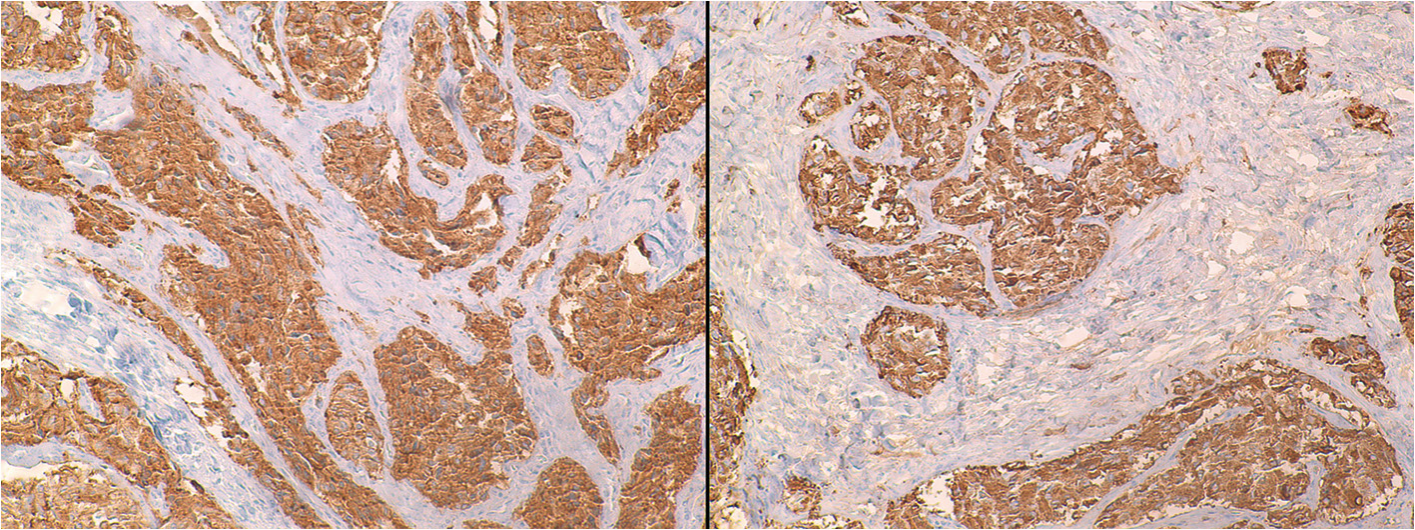
**Immunohistochemistry.** Photomicrographs of the specimen showing the tumor cells positive for synaptophysin, *left*, and chromogranin, *right*, which confirmed the diagnosis of carcinoid tumor.

Permanent tissue analysis of the specimen (hematoxylin and eosin) revealed sheet-like proliferation of neuroendocrine tumor cells in a trabecular pattern and no mitoses or necrosis (Figure 2), suggesting the diagnosis of carcinoid tumor. Special immunohistochemical staining revealed strong positivity for synaptophysin and chromogranin (Figure 3), which confirmed the diagnosis of carcinoid tumor.

**Figure 4:**
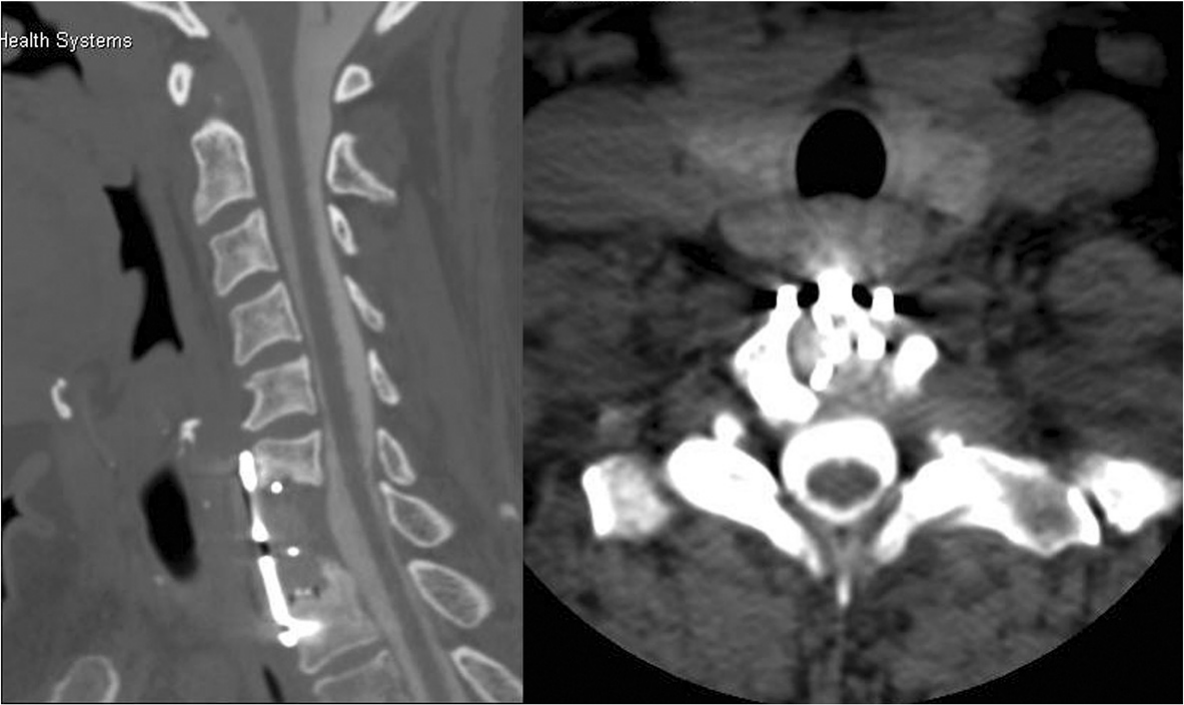
**Postoperative computed tomography-myelogram of the cervical spine.***Left*, sagittal and *right*, axial computed tomography-myelographic images illustrating the degree of bony removal (C7 corpectomy) and the complete decompression of the spinal cord and neural foramen.

Extensive postoperative work-up, including octreotide and technetium scintigraphy, as well as positron emission tomography scanning, failed to reveal any other carcinoid lesions elsewhere in the body. At the 6-year follow-up visit, the patient remains asymptomatic, with no evidence of local tumor recurrence (Figure 4), nor any other site for carcinoid tumors.

## Discussion

Carcinoid tumors arise from enterochromaffin cells and have an incidence of 0.28 to 0.8 per 100,000 per year [2]. When hormonally active, these tumors induce the carcinoid syndrome, characterized by flushing, diarrhea, vasodilation, bronchoconstriction, and other symptoms of increased serotonin release. More often, spinal carcinoid tumors manifest by mass effect: myelopathy and/or radiculopathy [6-8,13-15]. The histopathological appearance is that of a neuroendocrine tumor, with nests and trabeculae of cells exhibiting round or oval nuclei and rare mitoses [16]. The definitive diagnosis is based on positive immunohistochemical [16] staining for chromogranin A and synaptophysin.

There have been several reports of carcinoid metastases to the cervical spine. A 51-year-old man with a history of gastric carcinoid and multiple endocrine neoplasias presented with symptoms of myelopathy, and a cervical MRI revealed an osteoblastic tumor arising from the posterior arch of C2, compressing the spinal cord. The authors performed a surgical removal of the tumor, followed by radiation [8]. Another young patient with known intestinal carcinoid tumor presented with neck pain and was diagnosed with an osteolytic lesion in the left lateral mass of C1, threatening to erode into the vertebral canal. The authors described an elegant CT-guided vertebroplasty treatment of the lesion, with complete resolution of symptoms [5].

Our patient presented with similar symptoms of myelopathy and radiculopathy. The MRI confirmed the presence of an extradural tumor compressing the spinal cord and left C7 spinal nerve. However, there was no clinical or radiological evidence of a primary tumor that would make metastasis likely, and there were no signs of hormonal imbalance to suggest a neuroendocrine tumor. Therefore, the surgical intervention was started with a wide differential diagnosis in mind and with the decision of total versus partial resection to be made based on intraoperative findings, that is, the amount of tumor involvement with the neural elements. Because the tumor could be relatively easily separated from the dura of the spinal cord and nerve, we performed a gross total resection, at the cost of removing a large portion of the C7 vertebral body. This, in turn, created the need for instrumented stabilization of the lower cervical spine: a C6–T1 fixation. Because there has been no local recurrence at the 6-year follow-up visit, we conclude that the surgical resection was curative. Moreover, because there has been no other manifestation of carcinoid tumor in this patient, it is probable that the cervical involvement was primary, not a metastasis from common sites, such as the gastrointestinal tract or bronchi.

## Conclusions

Primary cervical carcinoid tumor is a rare entity, but should be included in the differential diagnosis of enhancing expansile extradural masses compressing the spinal cord and nerves. Surgical intervention should aim at gross total resection, because it may provide a definitive cure. Spinal instrumentation may be needed to provide stability following tumor resection.

## Consent

Written informed consent was obtained from the patient for publication of this case report and accompanying images. A copy of the written consent is available for review by the Editor-in-Chief of this journal.

## Competing interests

The authors declare that they have no competing interests.

## Authors’ contributions

GT performed the operation, all authors discussed and analyzed the patient data; MN and DS were major contributors in writing the manuscript; all authors have read and approved the final manuscript.
